# Implementation of government-directed policy in the hospital setting: a modified Delphi study

**DOI:** 10.1186/s12961-019-0500-8

**Published:** 2019-11-27

**Authors:** S. M. Havers, E. Martin, A. Wilson, L. Hall

**Affiliations:** 1 School of Public Health and Social Work, Institute of Health and Biomedical Innovation, Queensland University of Technology, 60 Musk Ave, Kelvin Grove, QLD 4059 Australia; 2 Menzies Centre for Health Policy, University of Sydney, Sydney, NSW Australia; 3 School of Public Health, University of Queensland, Herston, QLD Australia

**Keywords:** Policy development, policy implementation, implementation research, policy research, government regulation, financial penalty, pay-for-performance, modified Delphi, implementation framework, feasibility

## Abstract

**Background:**

In the last 20 years governments have sought to introduce policy that improves the quality of care provided in hospitals, yet little research has been done to understand how these policies are implemented, factors that affect the implementation process or what should be considered by decision-makers during policy development or implementation planning. Experts with real-life experience in the introduction and implementation of policy are best placed to provide valuable insight into practical issues that affect implementation and the associated outcomes of these policies.

**Methods:**

A modified Delphi study of experts in hospital policy development and implementation was undertaken to investigate factors influencing the implementation of government-directed policy in the hospital setting. This study built on the findings of two previous studies — a qualitative study of clinician perspectives of policy implementation and a systematic review and meta-synthesis, in which common contextual factors and policy characteristics associated with policy implementation were ascertained. International experts with extensive experience in government-directed policy implementation at global, national, corporate, jurisdictional and organisational levels were asked to provide opinions on predetermined factors and the feasibility of considering these in policy development and implementation planning. Survey design and analysis was guided by the Consolidated Framework for Implementation Research.

**Results:**

Eleven experts from four countries and with different health system perspectives participated in the study. Consensus was reached on the importance of all predetermined factors in the first survey round with additional factors for investigation highlighted by participants for examination in subsequent rounds. On study completion, expert consensus was reached on 24 factors of importance; only 20 of these factors reached consensus for feasibility.

**Conclusions:**

Study findings indicated that, whilst there are multiple factors of importance in policy implementation across all Consolidated Framework for Implementation Research domains, some factors, such as establishment of roles and responsibilities for implementation and organisational lines of accountability, are feasible for consideration at a hospital level only. In addition, four factors did not reach consensus in terms of feasibility, indicating that it may not be practical to consider all factors of importance when implementing policy; this has important implications for implementation planning and resource allocation.

## Contributions to the literature

- Dissemination and adoption of interventions in the hospital setting has been readily described in the literature but little research has focused specifically on the implementation of government-directed policy.
- A number of key factors associated with implementation of policy were identified. Many of these were related to the regulatory nature of government-directed policy or policy characteristics established at the time of development.
- This study highlights that implementation of government-directed policy is different from the adoption of other interventions. Greater understanding is needed if more effective implementation of these interventions is to be facilitated.

## Background

Since the identification of issues with quality of care in the late 20th century [1], governments have sought to improve the safety and quality of care provided in hospitals through policy [2]. Some examples of these policies have focused specifically on known practice gaps and identified areas of risk [3], whilst others have been more performance- or value-based initiatives rewarding increased efficiency or effectiveness in order to reduce ever-growing healthcare costs [4]. In theory, hospitals respond to these government directives by making the necessary changes to meet policy requirements, modifying practices and ultimately improving patient care [5]. However, in reality, the process of implementation is extremely challenging. As a result, the impact of these policies and their associated outcomes varies greatly [6, 7].

Whilst considerable efforts have been made to better understand implementation and the associated contextual factors over the last two decades [8], little of this research has focused specifically on the implementation of government-directed policy in the hospital setting. When policy outcomes are published, data is typically reported one dimensionally with little consideration or description of implementation [9]. There also remains a disconnect between policy implementation research and implementation science [10]. Policy development guidelines and analysis approaches do not typically account for the hospital setting, the complex clinical environment or the multiple health system levels involved in the development and implementation of government-directed policy [6].

Government-directed policy, as an intervention, is different compared to other safety and quality initiatives [11]. Health policy is constructed within a political context [12] and developed externally to the organisations that are then required to integrate changes at a patient care level. As a result, policy directives must be translated through multiple health system and organisational levels before reaching the patient — this involves many decision-makers and stakeholders throughout the implementation process and adds significant complexity to the relationships and networks involved [13]. Government-directed policy is also a mandated or regulated intervention, meaning its ‘adoption’ by the hospital in which it is to be implemented is not voluntary, which has been found to impact on acceptability [11].

Understanding these influences and the practical considerations of them in policy development and implementation planning is vital to improve policy outcomes and facilitate more effective and efficient policy design and translation. Given the complexity and the distinctiveness of government-directed policy implementation, opinions of experts with real-life experience in the development and implementation of government-directed policy were sought to establish the factors that influence implementation in the hospital setting using a modified Delphi technique.

## Methods

A two-round modified Delphi study of international experts in policy implementation was undertaken to establish expert consensus on the importance of factors on policy implementation in the hospital setting, and the feasibility of considering these factors during policy development and implementation phases. The Delphi technique is a research method where sequential surveys or questionnaires are used to gain individual expert opinion across a number of rounds, as a means of establishing consensus opinion across the group of participants [14]. Participants receive the results of each survey round analysis prior to the subsequent round as a means of feeding back the level of agreement achieved in each round. The benefits of this technique include the ability to gain the perspectives of a broadly experienced group of experts (in this case from multiple countries and healthcare systems) and build consensus in an area where relevant literature or evidence may be lacking [14, 15].

In this study, survey development, data collection, analyses and reporting of results were guided by the domains of the Consolidated Framework for Implementation Research (CFIR), a framework widely applied in the design and evaluation of implementation research [16]. The CFIR offered several advantages in terms of scientific rigor in the examination of factors of the importance of factors on policy implementation in the hospital setting, including domains that encompassed both external and internal health system structures, which were likely to be of importance in terms of government-directed policy implementation. Use of the CFIR for survey structure also aimed to facilitate greater participant understanding, and a more systematic approach to analysis across both survey rounds.

### Study participants

Participants were experts in government-directed policy development or implementation to ensure consensus was grounded in an applied understanding of policy implementation in the complex environment of the hospital setting.

Purposive and snowball sampling methods were used to recruit participants who had previously been involved in the development and/or implementation of government-directed policy in at least one hospital or more in a high-income country (as defined by World Bank Analytical Classifications [17]). A list of potential participants was initially established based on professional contacts of each member of the research team or professional networks of which one (or more) of the research team was a member. Guided by the methods described by Hasson et al. [18], purposeful sampling focused on the recruitment of experts with multi-level perspectives and real-life implementation experience rather than a large sample size; as such, recruitment emails were sent to the individually identified potential participants. Twenty-three participants were initially purposively contacted with an additional seven potential participants contacted as a result of snowball sampling. Experience in policy development or implementation at a global, national, jurisdictional or hospital level were all considered to be relevant. Experience in a specific health policy area was not required in order to broaden participant perspectives and maximise the scope of findings. Overall, 30 potential participants from Australia, the United States, the United Kingdom, Israel and a global policy body were contacted directly by email and provided with a short introductory communication that included study background and selection criteria.

### Ethical review

Ethical approval was gained through the Queensland University of Technology Human Research Ethics Committee (approval number 1800000778).

### Survey design

Factors for examination were constructed based on the results of two previously published studies. In the first study (a qualitative study of clinician perspectives of policy implementation), seven common contextual factors for policy implementation were found [11]. The second study involved a systematic review and meta-synthesis of all literature where implementation of government-directed health policy was described to identify common characteristics associated with policy implementation (PROSPERO Registration number CRD42018108123, https://www.crd.york.ac.uk/prospero/). From this study, three overarching intervention characteristic themes and 16 related sub-themes were identified.

Where common factors were identified across the two studies, these factors were amalgamated, resulting in a final list of 19 pre-determined factors. One factor regarding skill and competency assessment identified in the first study was determined to be encompassed within the factor of 'methods for monitoring and reporting implementation', so was not considered to be a seperate factor for investigation. The predetermined factors were then grouped into the five domains of the CFIR, namely policy (intervention) characteristics, outer setting, inner setting, characteristics of individuals and process (Table 1). Wording of the survey questions and how the participants were asked to consider the factors was developed directly from the previous studies’ findings, particularly in regard to the questions referring to clinician perception and alignment.

**Table 1:** Predetermined factors and associated domains used in round 1 of the modified Delphi survey

Factor	
Policy characteristics	
Perception of the quality and validity of the evidence	
Policy content and requirements/actions	
Valid and reliable evidence for policy requirements/actions	
Cost of implementation	
Outer setting	
External systems and infrastructure	
Alignment with national/corporate goals	
Regulatory mechanisms (e.g. accreditation, financial penalties)	
Inner setting	
Internal systems and infrastructure	
Established roles and responsibilities	
Internal relationships	
Organisational lines of accountability	
Methods for monitoring and reporting policy implementation outcomes	
Organisational stability	
Organisational goal setting	
Individuals	
Resources and tools	
Integration of policy requirements into current practice	
Implementation process	
Resourcing and preparedness	
Development of implementation plan	
Establishment of capacity in implementation planning	

Participants were asked to provide responses on the importance of each of the predetermined factors. To gather valuable information about the practicalities of considering these factors in policy development and implementation planning, participants were also asked about feasibility.

Responses were collected by asking participants to provide an indication of agreement with each of the statements on importance and feasibility using a five-point Likert scale [19]. An open text box for additional comments was provided for each factor and participants were requested to provide additional comments, information or perspectives related to the specific factor as well as suggestions for factors requiring further investigation in the subsequent round (Additional file 1).

In the subsequent survey round, participants were provided with a summary of the responses from the first round for each factor. For factors where consensus had not been met, participants were asked to review again and give a rating. In response to participant comments, additional factors were added and some questions rephrased in round 2 (Additional file 2). An open text box for comments was not included in the second survey given this round was focused on gaining perspectives on the findings of the previous round and any changes in consensus levels of the factors identified, both previously and in the first round of survey.

### Data collection

Participant’s responses were collected using a secure online survey programme (KeySurvey). For the first survey round, participants accessed the survey by a link included in the introductory email and were required to complete a Statement of Consent to progress to the survey questions. In the subsequent round, a separate web link was sent directly by email to the participant. Participants were given 2 weeks to complete each survey round. One reminder was sent to participants who had not completed the survey during the 2-week period to maximise the number of responses. Surveys were subsequently closed to allow analysis before the opening of the subsequent survey round. Each survey round took approximately 20 min to complete.

### Analysis

An analysis of responses was performed at the completion of each survey round. As there is no universally defined level of agreement for consensus for surveys using the modified Delphi method [18], the decision was made to determine that consensus had been met if scores of ‘very important’ and ‘important’ combined with those of ‘very feasible’ and ‘feasible’ were >70%. Consensus was determined as ‘not met’ if consensus was <70% for the same factor for two consecutive rounds (in either importance or feasibility scores). In addition, the first-round analysis included a thematic analysis of the open text comments to identify additional factors for inclusion in the subsequent round or rephrasing of questions as required.

## Results

### Round 1

Eleven of the 30 potential participants responded to the first survey round. Participant demographics and professional background are presented in Table 2. All participants indicated that they had been involved in both policy development and implementation. Average length of time working in this field was 17.1 years.

**Table 2:** Participant demographics

Demographic	Frequency	Percentage
Country		
Australia	6	54.5
United Kingdom	2	18.2
Israel	2	18.2
Global (multi-country)	1	9.1
Policy experience		
Development	11	100.0
Implementation	11	100.0
Years of experience		
5–10 years	2	18.2
11–15 years	4	36.4
16–20 years	3	27.3
20–30 years	1	9.1
30+ years	1	9.1
Current role		
Academic/researcher	3	27.3
Hospital/health services management		
- Organisation level role	1	9.1
- Jurisdiction/state level role	3	27.3
- National level role	3	27.3
Other (healthcare consultant)	1	9.1

The level of agreement for each factor is presented in Table 3. All 19 factors reached consensus in the first round for importance; however, only 13 of these factors reached consensus in terms of feasibility.

**Table 3:** Round 1 survey results for importance and feasibility of predetermined factors

Round 1	Level of agreement	
Factor	Importance	Feasibility
Policy characteristics		
Perception of the quality and validity of the evidence	90.9%	72.7%
Policy content and requirements/actions	100%	90.9%
Valid and reliable evidence for policy requirements/actions	100%	54.6%
Cost of implementation	100%	100%
Outer setting		
External systems and infrastructure	100%	72.7%
Alignment with national/corporate goals	90.9%	81.8%
Regulatory mechanisms (e.g. accreditation, financial penalties)	81.8%	90.9%
Inner setting		
Internal systems and infrastructure	90.9%	100%
Established roles and responsibilities	81.8%	63.6%
Internal relationships	100%	36.4%
Organisational lines of accountability	90.9%	63.6%
Methods for monitoring and reporting policy implementation outcomes	90.9%	72.7%
Organisational stability	90.9%	27.3%
Organisational goal setting	90.9%	72.7%
Individuals		
Resources and tools	81.8%	90.9%
Integration of policy requirements into current practice	100%	90.9%
Implementation process		
Resourcing and preparedness	90.9%	81.8%
Development of implementation plan	100%	72.7%
Establishment of capacity in implementation planning	72.7%	63.6%

Thematic analysis of open comments in the first round identified an additional five factors for examination. Additional factors were identified across four of the five domains, these included assessment and testing of policies for unforeseen circumstances and acceptability, clinician perception of policy benefits to patient care/outcomes, alignment of policy actions with other external requirements, clinical leaders/professional peers and the identification of changes to move from current practice to proposed practices.

In response to participant comments and lack of consensus, several questions were re-phrased. In the first instance, experts provided feedback on availability of evidence. As such, the question on feasibility associated with this factor was rephrased to include ‘when available’. Several of the experts also highlighted that, whilst important, some factors were only feasible for consideration at a hospital level, indicating that some factors may not be relevant at all policy development and implementation levels.

### Round 2

Nine of the 11 experts who initially completed the first survey round completed the second survey. The level of agreement for the second round is presented in Table 4. All additional factors identified in the first-round analysis reached consensus in the second round for importance, resulting in a total of 24 factors of importance for implementation from both rounds. Four of these factors did not reach consensus in terms of feasibility. Three of these were those factors that had been rephrased to be focused at a hospital level based on expert comments.

**Table 4:** Round 2 modified Delphi survey results for importance and feasibility of additional and rephrased factors

Survey questions	Level of agreement
Policy characteristics	
Where valid and reliable evidence is available, how feasible is it to ensure policy requirements/actions are supported by valid and reliable evidence when developing policy for implementation?	88.9%
Where valid and reliable evidence is not available, how important is it for proposed changes to be assessed/tested for unforeseen consequences and/or acceptability?	88.9%
Where valid and reliable evidence is not available, how feasible is it for proposed changes to be assessed/tested for unforeseen consequences and/or acceptability?	66.7%
How important is clinician perception that a policy will benefit patient care/outcomes for policy implementation?	77.8%
How feasible is it to consider clinician and decision-maker perception of whether a policy will benefit patient care/outcomes when developing policy for implementation?	88.9%
Outer setting	
How important is the alignment of policy actions and outcomes with other externally developed requirements for policy implementation?	100%
How feasible is it to ensure required policy actions and outcomes do not contradict other externally developed requirements during policy development?	88.9%
Inner setting	
How feasible is it for hospitals to consider roles and responsibilities before commencing policy implementation?	66.7%
How feasible is it for hospitals to undertake internal system and infrastructure assessment to identify potential local factors that influence policy implementation?	100%
How feasible is it for hospitals to establish organisational lines of accountability for implementation outcomes when developing policy for implementation?	100%
How feasible is it for hospitals to consider organisational stability when planning for policy implementation?	55.6%
How important are clinical leaders/professional peers (both formal and informal) in facilitating policy implementation?	100%
How feasible is it for hospitals to identify potential clinical leaders/professional peers that may influence implementation before commencing policy implementation?	88.9%
Implementation process	
How feasible is it for hospitals to quantify and build capacity for implementation before commencing policy implementation?	66.7%
How important is the identification of change/s required to move from current practice to proposed practice?	100%
How feasible is it to consider the change/s required to move from current practice to proposed practice and consider this transition when developing policy for implementation?	100%

#### Factors of importance

A list of all 24 factors are presented in Table 5 and described in relation to the CFIR domains below.

**Table 5:** Final list of factors of importance and indication of feasibility in policy implementation

Factor	Feasibility		
	Yes	Local level	No
Policy characteristics			
Perception of the quality and validity of the evidence	✓		
Policy content and requirements/actions	✓		
Valid and reliable evidence for policy requirements/actions	✓		
Assessment/testing of policy			x
Perception of benefit to patient care outcomes	✓		
Cost of implementation	✓		
Outer setting			
External systems and infrastructure	✓		
Alignment of policy with other external requirements	✓		
Alignment with national/corporate goals	✓		
Regulatory mechanisms (e.g. accreditation, financial penalties)	✓		
Inner setting			
Internal systems and infrastructure	✓		
Hospitals establish roles and responsibilities			x
Hospitals assess for internal factors/relationships		✓	
Hospitals establish organisational lines of accountability		✓	
Hospitals consider organisational stability			x
Methods for monitoring and reporting policy implementation outcomes	✓		
Clinical leaders/professional peers		✓	
Organisational goal setting		✓	
Individuals			
Resources and tools	✓		
Integration of policy requirements into current practice	✓		
Implementation process			
Resourcing and preparedness	✓		
Development of implementation plan	✓		
Hospitals establish and build capacity for implementation			x
Identification of changes needed in planning	✓		

Six factors of importance were associated with the domain of ‘Policy characteristics’. Acceptability and perception of policy content were commonly discussed by experts when responding to elements in this domain, an important finding with implications for policy developers given policy content is predominantly developed by the government or associated entity, external to the hospital setting and clinicians. Four factors were included in the outer setting and eight in the inner setting; the factors within the outer and inner settings (and the potential interactions across the two domains) indicate the complexity of relationships that exist when attempting to transition government-directed policy to the clinical environment and are reflective of other barriers and facilitators commonly described in change management and organisational behaviour theory literature [20].

Only two factors associated within the ‘Individuals’ domain were identified with no additional factors added, highlighting the role of system and infrastructure factors rather than individual behaviour change processes in policy implementation. Factors associated with the final domain of implementation planning were focused on the development of an implementation plan, capacity and resourcing, and identification of changes needed. Although several experts indicated that implementation capacity and resourcing should be considered at a hospital level, this factor did not reach consensus in terms of feasibility of consideration.

#### Feasibility

Asking participants to comment on feasibility provided important practical insights, with the findings identifying the differences in feasibility across the different levels of the health system. As noted, several of the factors were described to only be feasible for consideration at a local level. Assessment of internal structures and relationships was one of these factors.

“*The feasibility question is difficult given that* [these] *internal system and structures generally do not support implementation in any effective way.*” (Participant 10) 

Study findings also highlighted the practical challenges involved in translating government-directed policies across multiple health systems from “*work as imagined*” into real-life, “*work as done*” [21], as one expert commented in regards to ensuring policy content clarity:

“*It is often very poorly done because those writing the policy do not have a clear understanding of how work is actually delivered and how policy is likely to be interpreted at the coal face.*” (Participant 10)

In addition, consensus on the feasibility of certain factors demonstrated that some factors, whilst important and likely to influence implementation, may not be able to be considered or controlled.

An example of this was the factor of organisational stability. Although expert consensus demonstrated the importance of this factor, several participants highlighted that changing this is unlikely to be part of policy implementation or planning.

“*It is important from an implementation point of view because a stable organisation is more likely to be able to implement necessary change. It is not feasible though to consider this when developing a policy as this can change so quickly so you need to assume that each health service is stable.*”  (Participant 1)

This is an important lesson in terms of resource investment and focusing efforts in implementation on factors that are both important and feasible.

From a practical perspective, this study also reaffirmed issues with the availability of valid and reliable data. Again, this phenomenon is well-described in the literature [22]; however, study participants indicated that acceptability was not only influenced by valid evidence and, whilst it should be considered where available, policy acceptability and clinician perspectives of policy, in particular, are influenced by several factors, evidence being only one of these.

“*Evidence from peer reviewed literature is important, although even if the evidence is strong, if it does not match with the core beliefs of the clinician, it will not be sufficient to drive behaviour change.*” (Participant 11)

“*Gaining traction with policy changes that do not directly benefit the patient is more challenging irrespective of the strength of the evidence.*” (Participant 2)

Acceptability was also highlighted in the addition of several factors to do with policy testing and assessment and clinician perception of patient benefit.

“*In healthcare, more so than other fields, there is a need for the evidence to ‘tell a story’ that leads to improved patient outcomes through the proposed change.*” (Participant 2)

Factors that did not reach consensus provide additional insight and an indication of where future research may be of value. Although all factors reached consensus from an importance perspective, two of the factors that did not reach consensus in terms of feasibility were associated with policy implementation processes; these were testing and assessment of policy and establishing and building capacity for implementation.

“*This is dependent on how the organisation views implementation and how many resources are provided. There is generally no further capacity for implementation.*” (Participant 2)

Given this scarcity of resources and limited capacity, it is possible that the feasibility in planning and capacity-building for implementation are impacted on by the limited research in this field and how best to approach these in the planning process. Further research that leads to firm recommendations in terms of implementation planning and evaluation would be highly valuable.

Lastly, three of the four factors of importance that were found not to be feasible were relevant at a hospital level. It is possible that resource scarcity in the hospital environment influences the feasibility of the factors being considered. This would also support findings of previous studies that showed the difficulties with requiring front-line clinicians to implement policy at a hospital level in a complex and busy setting with little to no supporting infrastructure [11].

## Discussion

Use of the modified Delphi approach in this study enabled an in-depth and pragmatic examination of factors that influence the implementation of government-directed policy in the hospital setting, combining the opinions and perspectives of experts with real-life policy development and implementation experience. Purposive sampling enabled recruitment of participants from different countries, health systems and policy development and implementation perspectives, improving content validity. Providing participants with a summary of findings from the first round may also have potentially challenged each individual participant to reassess their perspective prior to responding to the subsequent round [23] as well as prompting further consideration of both the predetermined and newly identified factors in the second round. Final analysis of the study findings showed consensus was reached on 24 factors of importance in policy development and implementation across all the CFIR domains, with 20 of these also found to be feasible for consideration during policy development and implementation, although some only at a hospital level.

The study findings, particularly the number of factors identified, the differences between importance and feasibility, and the identification of several factors of importance only for consideration at an organisational level, highlights the complexities and challenges faced by those tasked with the development and implementation of government-directed policy in the hospital setting and the influence of the multiple health system levels on implementation processes. This complexity and the multiple and interacting factors may also provide some indication of why the impact of government policies on healthcare quality or patient outcomes remains highly variable [6, 24, 25]. With so many factors of influence in the implementation of government-directed policies it is not surprising that policies may not achieve their intended outcomes. Although some examples of government policy have been shown to have had significant impact on patient care and outcomes [26], other substantial pieces of policy (even at a national level) have been criticised for their minimal overall impact [27, 28].

The results of this study also demonstrate that a simple linear progression of the policy process is not appropriate. It is not effective for governments to simply establish a particular agenda or policy intention, develop a policy response and expect policy to be implemented [12, 29]. There is growing recognition that this simplistic approach to policy development does not adequately capture the challenges in achieving policy goals [30] or the realities of policy development and implementation in the complex and highly dynamic healthcare setting [26, 31, 32], and that this inadequate understanding of policy implementation is impacting poorly on the achievement policy outcomes [32]; this study and the consensus established certainly supports this.

The feasibility of some of the factors assessed in the Delphi is important to consider. For example, the assessment of internal structures and relationships was deemed only feasible at a hospital level, and some factors were not feasible for consideration at all such as understanding organisational stability. This is not surprising given that the hospital environment itself presents a unique environment for implementation. Organisational factors are commonly identified as having a significant influence on the implementation of safety and quality interventions and the associated decision-making processes of individuals within a healthcare organisation [5, 16, 33–35]. Whilst many of these factors were identified to be of importance for policy implementation in this study, it was of interest that consideration of some of these factors was found to only be applicable at an organisational level. This delineation suggests that there are factors that should not be considered by policy developers or higher-level administrators at a jurisdictional or regional level. This greater understanding of the relevant factors and who might be best placed to consider them could possibly enable more efficient and clearly defined implementation planning roles and activities.

### Limitations

There are several limitations to the findings of this study. Firstly, the lack of participants from the United States, despite purposive sampling of potential participants from this country, impacts on the application of findings in a broader sense. This is of interest given the considerable differences in funding structures and performance-based policies introduced in the United States in the last 10 years. Whilst it would have been valuable to have expert opinion from this perspective, additional research comparing opinions from this study to a United States population study could possibly be more valuable and provide a richer insight into the differences in policy approaches from an implementation perspective. Another limitation was sample size, although our sample is in line with previously published Delphi survey recommendations [36]. It was a purposeful decision of the study team to limit the number of respondents to those with broader, real-life, ‘higher level’ perspectives, focusing on quality responses rather than a great number of respondents. The richness of comments and perspectives shared by participants in the first survey round suggest that this sampling approach was appropriate. Additionally, given that one of the studies from which the predetermined factors were identified collected perspectives at a clinician/hospital level, sampling was focused predominantly on global/national and jurisdictional level experts, thus limiting the number of potential participants.

## Conclusion

This study identified 24 factors of importance in policy implementation in the hospital setting and confirmed that these factors exist across multiple levels of the healthcare system and the policy development and implementation process. Importantly, expert opinion also highlighted that consideration of all factors of importance may not be feasible during development and planning or may only be relevant at a local hospital level. Further research is needed to understand how consideration of factors of importance should be integrated into policy development and implementation processes. Such research would help guide policy-makers and decision-makers to develop policy that is more effectively and efficiently implemented and ultimately more likely to achieve the intended outcomes.

## Supplementary information

**Additional file 1.** Modified Delphi survey - Round 1

**Additional file 2.** Modified Delphi survey - Round 2

## Data Availability

The data that support the findings of this study are available on request from the corresponding author (SMH). The data are not publicly available due to them containing information that could compromise research participant privacy.

## Abbreviations

CFIR: Consolidated Framework for Implementation Research
