# Supplementary material for: Implementation of government-directed policy in the hospital setting: a modified Delphi study
Source: Health Res Policy Syst. 2019 Nov 27;17:91. doi: 10.1186/s12961-019-0500-8 (PMC6880558; doi:10.1186/s12961-019-0500-8)
Supplement: Supplementary file 1 — Additional file 1. Modified Delphi survey - Round 1 [file 12961_2019_500_MOESM1_ESM.pdf]

Expert consensus on the factors that influence policy implementation in the hospital setting

The purpose of this survey is to establish consensus on factors that influence policy implementation in the hospital setting. The questions will be administered by online survey and will take approximately 20 minutes to complete.

QUT Ethics Approval Number: 1800000778 RESEARCH TEAM Principal Researcher: Sally Havers Associate Researcher: Dr. Elizabeth Martin Faculty of Health Thank you for helping with this research project.

Your participation in this project is entirely voluntary. If you do agree to participate you can withdraw from the project during the survey round without comment or penalty by closing the survey window. You will not be able to complete the survey unless you consent to do so. By clicking on the "Next" button below, you are indicating that you: • Have read and understood the information document regarding this project. • Have had any questions answered to your satisfaction. • Understand that if you have any additional questions you can contact the research team. • Understand that you are free to withdraw at any time without comment or penalty. • Understand that if you have concerns about the ethical conduct of the project you can contact the Research Ethics Advisory Team on 07 3138 5123 or email humanethics@qut.edu.au. • Understand that non-identifiable data collected in this project may be used as comparative data in future projects. • Agree to participate in the project. Click "Next" to start survey

SECTION B: Participant Demographics Please provide the following demographics:

1. Country:

- ○ Australia
- ○ United States
- ○ United Kingdom
- ○ Israel
- ○ Other (please specify below):

....

2. Qualification/s:

....
....
....
....

3. Current Role/Job Title:

....
....
....
....

4. Have you been involved in policy development?

- ○ Yes
- ○ No

5. Have you been involved in policy implementation?

- ○ Yes
- ○ No

6. Length of time (in years) working in policy development and/or implementation:

....

....

....

....

SECTION C: Factors that influence policy implementation

POLICY CHARACTERISTICS

7a. How important is the perception of the quality and validity of the evidence referred to/cited in policy documentation for policy implementation?

- ○ Very Important
- ○ Important
- ○ Neutral
- ○ Unimportant
- ○ Highly Unimportant

7b. How feasible is it to consider how clinicians and decision makers perceive the quality and validity of evidence available when developing policy for implementation?

- ○ Highly Feasible
- ○ Feasible
- ○ Neutral
- ○ Not Feasible
- ○ Really Not Feasible

Please provide any general comments you have on the perception of quality and validity of evidence available in policy implementation:

....

....

....

....

8a. How important is policy content and requirements/actions for policy implementation?

- ○ Very Important
- ○ Important
- ○ Neutral
- ○ Unimportant
- ○ Highly Unimportant

8b. How feasible is it to ensure policy content and requirements/actions are clear when developing policy for implementation?

- ○ Highly Feasible
- ○ Feasible
- ○ Neutral
- ○ Not Feasible
- ○ Really Not Feasible

Please provide any general comments you have on policy content and clarity of requirements/actions in policy implementation:

....

....

....

....

9a. How important is valid and reliable evidence for policy requirements/actions for policy implementation?

- ○ Very Important
- ○ Important
- ○ Neutral
- ○ Unimportant
- ○ Highly Unimportant

9b. How feasible is it to ensure policy requirements/actions are supported by valid and reliable evidence when developing policy for implementation?

- ○ Highly Feasible
- ○ Feasible
- ○ Neutral
- ○ Not Feasible
- ○ Really Not Feasible

Please provide any general comments you have on policy requirements/actions being supported by valid and reliable evidence for policy implementation:

....

....

....

....

10a. How important is the cost of implementation for policy implementation?

- ○ Very Important
- ○ Important
- ○ Neutral
- ○ Unimportant
- ○ Highly Unimportant

10b. How feasible is it to consider the cost of implementation when developing policy for implementation?

- ○ Highly Feasible
- ○ Feasible
- ○ Neutral
- ○ Not Feasible
- ○ Really Not Feasible

Please provide any general comments you have related to cost of implementation and policy implementation:

....

....

....

....

SECTION C: Factors that influence policy implementation

OUTER SETTING

11a. How important are external systems and infrastructure (including health system level structures) for policy implementation?

- ○ Very Important
- ○ Important
- ○ Neutral
- ○ Unimportant
- ○ Highly Unimportant

11b. How feasible is it to consider the role of external systems and infrastructure (including health system level structures) when developing policy for implementation?

- ○ Highly Feasible
- ○ Feasible
- ○ Neutral
- ○ Not Feasible
- ○ Really Not Feasible

Please provide any general comments you have related to external systems and infrastructure (including health system level structures) in policy implementation:

....

....

....

....

12a. How important is alignment with national or corporate goals, visions and/or strategies (e.g. at a health system or corporate level) for policy implementation?

- ○ Very Important
- ○ Important
- ○ Neutral
- ○ Unimportant
- ○ Highly Unimportant

12b. How feasible is it to consider external goal setting (e.g. at a health system or corporate level) when developing policy for implementation?

- ○ Highly Feasible
- ○ Feasible
- ○ Neutral
- ○ Not Feasible
- ○ Really Not Feasible

Please provide any general comments you have related to external goal setting (e.g. at a health system or corporate level) in policy implementation:

....

....

....

....

13a. How important are regulatory mechanisms (for example - external reviews or accreditation, financial penalties, licencing restrictions) for policy implementation?

- ○ Very Important
- ○ Important
- ○ Neutral
- ○ Unimportant
- ○ Highly Unimportant

13b. How feasible is it to consider regulatory mechanisms (for example - external reviews or accreditation, financial penalties, licencing restrictions) when developing policy for implementation?

- ○ Highly Feasible
- ○ Feasible
- ○ Neutral
- ○ Not Feasible
- ○ Really Not Feasible

Please provide any general comments you have related to regulatory mechanisms (for example - external reviews or accreditation, financial penalties, licencing restrictions) in policy implementation:

....

....

....

....

SECTION C: Factors that influence policy implementation

INNER SETTING

(PLEASE NOTE FOR Q14a & b - Internal systems and infrastructure means the establishment of organisational systems, processes and functionality that does not rely on clinicians providing direct patient care to undertake implementation.) 14a. How important are internal systems and infrastructure* to support implementation for policy implementation?

- ○ Very Important
- ○ Important
- ○ Neutral
- ○ Unimportant
- ○ Highly Unimportant

14b. How feasible is it to consider internal systems and infrastructure requirements when developing policy for implementation?

- ○ Highly Feasible
- ○ Feasible
- ○ Neutral
- ○ Not Feasible
- ○ Really Not Feasible

Please provide any general comments you have related to internal systems and infrastructure in policy implementation:

....

....

....

....

15a. How important are established roles and responsibilities for individual clinicians and decision makers in policy implementation?

- ○ Very Important
- ○ Important
- ○ Neutral
- ○ Unimportant
- ○ Highly Unimportant

15b. How feasible is it to consider roles and responsibilities when developing policy for implementation?

- O Highly Feasible
- O Feasible
- O Neutral
- O Not Feasible
- O Really Not Feasible

Please provide any general comments you have related to roles and responsibilities for policy implementation:

....

....

....

....

16a. How important are are internal relationships for policy implementation?

- O Very Important
- O Important
- O Neutral
- O Unimportant
- O Highly Unimportant

16b. How feasible is it to consider internal relationships when developing policy for implementation?

- O Highly Feasible
- O Feasible
- O Neutral
- O Not Feasible
- O Really Not Feasible

Please provide any general comments you have related to internal relationships for policy implementation:

....

....

....

....

17a. How important are established organisational lines of accountability for implementation outcomes for policy implementation?

- O Very Important
- O Important
- O Neutral
- O Unimportant
- O Highly Unimportant

17b. How feasible is it to consider organisational lines of accountability for implementation outcomes when developing policy for implementation?

- O Highly Feasible
- O Feasible
- O Neutral
- O Not Feasible
- O Really Not Feasible

Please provide any general comments you have related to organisational lines of accountability in policy implementation:

....

....

....

....

18a. How important are the methods for monitoring and reporting policy implementation outcomes?

- O Very Important
- O Important
- O Neutral
- O Unimportant
- O Highly Unimportant

18b. How feasible is it to consider methods for monitoring and reporting policy implementation outcomes when developing policy for implementation?

- ○ Highly Feasible
- ○ Feasible
- ○ Neutral
- ○ Not Feasible
- ○ Really Not Feasible

Please provide any general comments you have related to methods for monitoring and reporting policy implementation outcomes:

....

....

....

....

19a. How important is organisational stability for policy implementation?

- ○ Very Important
- ○ Important
- ○ Neutral
- ○ Unimportant
- ○ Highly Unimportant

19b. How feasible is it to consider organisational stability when developing policy for implementation?

- ○ Highly Feasible
- ○ Feasible
- ○ Neutral
- ○ Not Feasible
- ○ Really Not Feasible

Please provide any general comments you have related to organisational stability and policy implementation:

....

....

....

....

20a. How important is organisational goal setting for policy implementation?

- O Very Important
- O Important
- O Neutral
- O Unimportant
- O Highly Unimportant

20b. How feasible is it to consider organisational goal setting when developing policy for implementation?

- O Highly Feasible
- O Feasible
- O Neutral
- O Not Feasible
- O Really Not Feasible

Please provide any general comments you have related to organisational goal setting and policy implementation:

....

....

....

....

SECTION C: Factors that influence policy implementation

INDIVIDUALS

21a. How important are resources and tools in changing individual behavior as a result of policy implementation?

- O Very Important
- O Important
- O Neutral
- O Unimportant
- O Highly Unimportant

21b. How feasible is it to consider the development of resources and tools in changing individual behavior when developing policy for implementation?

- Highly Feasible
- Feasible
- Neutral
- Not Feasible
- Really Not Feasible

Please provide any general comments related to the development of resources and tools in changing individual behavior in policy implementation?

....
....
....
....

22a. How important is integration of policy requirements into current practices/patient care activities and workflow for policy implementation?

- Very Important
- Important
- Neutral
- Unimportant
- Highly Unimportant

22b. How feasible is it to consider the integration of policy into current practices/patient care activities and workflow when developing policy for implementation?

- Highly Feasible
- Feasible
- Neutral
- Not Feasible
- Really Not Feasible

Please provide any general comments you have related to the integration of policy into current practices/patient care activities and workflow in policy implementation?

....
....
....
....

SECTION C: Factors that influence policy implementation

IMPLEMENTATION PROCESS

23a. How important is resourcing and preparedness for policy implementation?

- ○ Very Important
- ○ Important
- ○ Neutral
- ○ Unimportant
- ○ Highly Unimportant

23b. How feasible is it to consider resourcing and preparedness when developing policy for implementation?

- ○ Highly Feasible
- ○ Feasible
- ○ Neutral
- ○ Not Feasible
- ○ Really Not Feasible

Please provide any general comments you have related to resourcing and preparedness in policy implementation?

....

....

....

....

24a. How important is development of a plan for implementation for policy implementation?

- ○ Very Important
- ○ Important
- ○ Neutral
- ○ Unimportant
- ○ Highly Unimportant

24b. How feasible is it to consider the development of an implementation plan when developing policy for implementation?

- Highly Feasible
- Feasible
- Neutral
- Not Feasible
- Really Not Feasible

Please provide any general comments you have related to development of an implementation plan in policy implementation?

....

....

....

....

25a. How important is it to establish capacity for implementation and resource implications when planning policy implementation?

- Very Important
- Important
- Neutral
- Unimportant
- Highly Unimportant

25b. How feasible is it to consider (and quantify) the capacity for implementation and resource implications when developing policy for implementation?

- Highly Feasible
- Feasible
- Neutral
- Not Feasible
- Really Not Feasible

Please provide any general comments you have related to capacity for implementation and resource implications in policy implementation?

....

....

....

....

Thank you for your valuable time. This survey will close after two weeks. Analysis of the results will be performed and a subsequent survey sent to all participants two weeks following. If you have any questions or concerns regarding the survey please do not hesitate to get in contact on the details provided at the start of this survey or in the Information Sheet provided.
