# Supplementary material for: Implementation of government-directed policy in the hospital setting: a modified Delphi study
Source: Health Res Policy Syst. 2019 Nov 27;17:91. doi: 10.1186/s12961-019-0500-8 (PMC6880558; doi:10.1186/s12961-019-0500-8)
Supplement: Supplementary file 2 — Additional file 2. Modified Delphi survey - Round 2 [file 12961_2019_500_MOESM2_ESM.pdf]

Round 2 - Expert consensus on the factors that influence policy implementation in the hospital setting

The purpose of this survey is to establish consensus on factors that influence policy implementation in the hospital setting. The questions will be administered by online survey and will take approximately 20 minutes to complete.

QUT Ethics Approval Number: 1800000778 RESEARCH TEAM Principal Researcher: Sally Havers Associate Researcher: Dr. Elizabeth Martin Faculty of Health Thank you for helping with this research project.

SECTION A: CONSENT REMINDER Your participation in this project is entirely voluntary. If you do agree to participate you can withdraw from the project during the survey round without comment or penalty by closing the survey window. You will not be able to complete the survey unless you consent to do so. Click "Next" to start survey

SECTION B: Factors that influence policy implementation

POLICY CHARACTERISTICS THE FOLLOWING QUESTIONS REACHED CONSENSUS IN ROUND 1 & DO NOT NEED TO BE ANSWERED AGAIN

How important is the perception of the quality and validity of the evidence referred to/cited in policy documentation for policy implementation?

How feasible is it to consider how clinicians and decision makers perceive the quality and validity of evidence available when developing policy for implementation?

How important is policy content and requirements/actions for policy implementation?

How feasible is it to ensure policy content and requirements/actions are clear when developing policy for implementation?

How important is valid and reliable evidence for policy requirements/actions for policy implementation?

How important is the cost of implementation for policy implementation?

How feasible is it to consider the cost of implementation when developing policy for implementation?

THE FOLLOWING QUESTION DID NOT REACH CONSENSUS - PLEASE SEE RESULTS BELOW How feasible is it to ensure policy requirements/actions are supported by valid and reliable evidence when developing policy for implementation? Response percent Highly Feasible 9.09% Feasible 45.46% Neutral 36.36% Not Feasible 9.09% Really Not Feasible 0%

Participant comments highlighted that valid and reliable evidence is often not available. Please see additional questions to address this: Where valid and reliable evidence is available, how feasible is it to ensure policy requirements/actions are supported by valid and reliable evidence when developing policy for implementation?

- ○ Highly Feasible
- ○ Feasible
- ○ Neutral
- ○ Not Feasible
- ○ Really Not Feasible

Where valid and reliable evidence is not available, how important is it for proposed changes to be assessed/tested for unforeseen consequences and/or acceptability?

- ○ Highly Important
- ○ Important
- ○ Neutral
- ○ Not Important
- ○ Really Not Important

Where valid and reliable evidence is not available, how feasible is it for proposed changes to be assessed/tested for unforeseen consequences and/or acceptability?

- ○ Highly Feasible
- ○ Feasible
- ○ Neutral
- ○ Not Feasible
- ○ Really Not Feasible

ADDITIONAL QUESTION BASED ON PARTICIPANT COMMENTS: How important is clinician perception that a policy will benefit patient care/outcomes for policy implementation?

- ○ Highly Important
- ○ Important
- ○ Neutral
- ○ Not Important
- ○ Really Not Important

ADDITIONAL QUESTION BASED ON PARTICIPANT COMMENTS: How feasible is it to consider clinician and decision maker perception of whether a policy will benefit patient care/outcomes when developing policy for implementation?

- Highly Feasible
- Feasible
- Neutral
- Not Feasible
- Really Not Feasible

SECTION B: Factors that influence policy implementation

OUTER SETTING THE FOLLOWING QUESTIONS REACHED CONSENSUS IN ROUND 1 & DO NOT NEED TO BE ANSWERED AGAIN

How important are external systems and infrastructure (including health system level structures) for policy implementation?

How feasible is it to consider the role of external systems and infrastructure (including health system level structures) when developing policy for implementation?

How important is alignment with national or corporate goals, visions and/or strategies (e.g. at a health system or corporate level) for policy implementation?

How feasible is it to consider external goal setting (e.g. at a health system or corporate level) when developing policy for implementation?

How important are regulatory mechanisms (for example - external reviews or accreditation, financial penalties, licencing restrictions) for policy implementation?

How feasible is it to consider regulatory mechanisms (for example - external reviews or accreditation, financial penalties, licencing restrictions) when developing policy for implementation?

OUTER SETTING THE FOLLOWING QUESTIONS HAVE BEEN ADDED AS A RESULT OF PARTICIPANT COMMENTS

How important is the alignment of policy actions and outcomes with other externally developed requirements for policy implementation? (This may include – other policies, jurisdictional performance monitoring, payment models etc.)

- Highly Important
- Important
- Neutral
- Not Important
- Really Not Important

How feasible is it to ensure required policy actions and outcomes do not contradict other externally developed requirements during policy development? (This may include – other policies, jurisdictional performance monitoring, payment models etc.).

- ○ Highly Feasible
- ○ Feasible
- ○ Neutral
- ○ Not Feasible
- ○ Really Not Feasible

SECTION C: Factors that influence policy implementation

INNER SETTING THE FOLLOWING QUESTIONS REACHED CONSENSUS IN ROUND 1 & DO NOT NEED TO BE ANSWERED AGAIN

How important are internal systems and infrastructure to support implementation for policy implementation?

How feasible is it to consider internal systems and infrastructure requirements when developing policy for implementation?

How important are established roles and responsibilities for individual clinicians and decision makers in policy implementation?

How important are are internal relationships for policy implementation?

How important are established organisational lines of accountability for implementation outcomes for policy implementation?

How important are the methods for monitoring and reporting policy implementation outcomes?

How feasible is it to consider methods for monitoring and reporting policy implementation outcomes when developing policy for implementation?

How important is organisational stability for policy implementation?

How important is organisational goal setting for policy implementation?

How feasible is it to consider organisational goal setting when developing policy for implementation?

THE FOLLOWING QUESTIONS DID NOT REACH CONSENSUS Participant comments indicated that consideration of INNER SETTING factors is potentially not feasible at the point of policy development but consideration of these factors should be facilitated at a hospital local level. These questions have been revised in response to these comments. Please see results and modified questions below: How feasible is it to consider roles and responsibilities when developing policy for implementation? Response percent Highly Feasible 18.18% Feasible 45.46% Neutral 27.27% Not Feasible 9.09% Really Not Feasible 0%

How feasible is it for hospitals to consider roles and responsibilities before commencing policy implementation?

- ○ Highly feasible
- ○ Feasible
- ○ Neutral
- ○ Not Feasible
- ○ Really Not Feasible

How feasible is it to consider internal relationships when developing policy for implementation? Response percent Highly Feasible 0.0% Feasible 36.36% Neutral 27.27% Not Feasible 36.36% Really Not Feasible 0%

How feasible is it for hospitals to undertake internal system and infrastructure assessment to identify potential local factors that influence policy implementation?

- ○ Highly Feasible
- ○ Feasible
- ○ Neutral
- ○ Not Feasible
- ○ Really Not Feasible

How feasible is it to consider organisational lines of accountability when developing policy for implementation? Response percent Highly Feasible 9.09% Feasible 54.55% Neutral 36.36% Not Feasible 0% Really Not Feasible 0%

How feasible is it for hospitals to establish organisational lines of accountability for implementation outcomes when developing policy for implementation?

- ○ Highly Feasible
- ○ Feasible
- ○ Neutral
- ○ Not Feasible
- ○ Really Not Feasible

How feasible is it to consider organisational stability when developing policy for implementation? Response percent Highly Feasible 0.00% Feasible 27.27% Neutral 45.46% Not Feasible 27.27% Really Not Feasible 0%

How feasible is it for hospitals to consider organisational stability when planning for policy implementation?

- Highly Feasible
- Feasible
- Neutral
- Not Feasible
- Really Not Feasible

INNER SETTING THE FOLLOWING QUESTIONS HAVE BEEN ADDED AS A RESULT OF PARTICIPANT COMMENTS

How important are clinical leaders/professional peers (both formal and informal) in facilitating policy implementation?

- Highly Important
- Important
- Neutral
- Not Important
- Really Not Important

How feasible is it for hospitals to identify potential clinical leaders/professional peers that may influence implementation before commencing policy implementation?

- Highly Feasible
- Feasible
- Neutral
- Not Feasible
- Really Not Feasible

SECTION B: Factors that influence policy implementation

INDIVIDUALS THE FOLLOWING QUESTIONS REACHED CONSENSUS IN ROUND 1 & DO NOT NEED TO BE ANSWERED AGAIN

How important are resources and tools in changing individual behavior as a result of policy implementation?

How feasible is it to consider the development of resources and tools in changing individual behavior when developing policy for implementation?

How important is integration of policy requirements into current practices/patient care activities and workflow for policy implementation?

How feasible is it to consider the integration of policy into current practices/patient care activities and workflow when developing policy for implementation?

SECTION B: Factors that influence policy implementation

IMPLEMENTATION PROCESS THE FOLLOWING QUESTIONS REACHED CONSENSUS IN ROUND 1 & DO NOT NEED TO BE ANSWERED AGAIN

How important is resourcing and preparedness for policy implementation?

How feasible is it to consider resourcing and preparedness when developing policy for implementation?

How important is development of a plan for implementation for policy implementation?

How feasible is it to consider the development of an implementation plan when developing policy for implementation?

How important is it to establish capacity for implementation and resource implications when planning policy implementation?

THE FOLLOWING QUESTION DID NOT REACH CONSENSUS - PLEASE SEE RESULTS BELOW How feasible is it to consider (and quantify) the capacity for implementation and resource implications when developing policy for implementation? Response percent Highly Feasible 9.09% Feasible 54.55% Neutral 27.27% Not Feasible 9.09% Really Not Feasible 0%

How feasible is it for hospitals to quantify and build capacity for implementation before commencing policy implementation?

- ○ Highly Feasible
- ○ Feasible
- ○ Neutral
- ○ Not Feasible
- ○ Really Not Feasible

ADDITIONAL QUESTION BASED ON PARTICIPANT COMMENTS: How important is the identification of change/s required to move from current practice to proposed practice and planning this transition for policy implementation?

- ○ Highly Important
- ○ Important
- ○ Neutral
- ○ Not Important
- ○ Really Not Important

ADDITIONAL QUESTION BASED ON PARTICIPANT COMMENTS: How feasible is it to consider the change/s required to move from current practice to proposed practice and consider this transition when developing policy for implementation?

- ○ Highly Feasible
- ○ Feasible
- ○ Neutral
- ○ Not Feasible
- ○ Really Not Feasible

Thank you for your valuable time completing this survey. If you have any questions or concerns regarding the survey please do not hesitate to get in contact on the details provided at the start of this survey or in the Information Sheet provided.
